# The Story of the Insane from Year to Year

**Published:** 1893-08-12

**Authors:** 


### THE STORY OF THE INSANE FROM YEAR TO YEAR.

(Fifth Series.)

### HEREFORD COUNTY ASYLUM.

This is the asylum for the county and city of Hereford, and is situated at Burghill. At the beginning of the year 376 patients were in residence, and at the end of December the numbers had fallen to 371. There were 81 admissions, 22 recoveries, and 34 deaths. The recoveries work out at 27·2 per cent. of the admissions, and the deaths at 8·9 per cent. on the average number resident. Only five of the admissions were owing to intemperance. Heredity, however, accounts for 15, and old age is given as the cause of the insanity in 18 cases. Two of the deaths were from consumption, and 16 from brain diseases. Dr. Chapman points out that although the number of patients was five fewer at the end than at the beginning of the year, such decrease was not owing to a decrease of admissions, but owing to the fact that ten patients were transferred to the Gloucester Asylum, which arrangement implies a loss of about £90 to the Hereford ratepayers. "Section 38 of the new Lunacy Act came into operation during the year, and led to the discharge of a few patients, in no case that I am aware of to their advantage, and in several distinctly to their injury. This and other clauses have added a good deal of extra, chiefly clerical, work, certainly without any benefit that I can discover to anyone, and, as I have stated, in some cases to the injury of patients. And I can easily imagine cases in which it might be to the injury of the public." So says Dr. Chapman, and he is well qualified to speak on the subject. The visitors "desire to express their appreciation of Dr. Chapman's services, and of the ability and diligence with which he performed all his varied duties." We are pleased to notice that the scale of wages has been raised, and that bonuses are now given to the attendants and nurses at the end of five years' services. This is a step in the right direction. Dr. Chapman can make farming pay. He has a balance of £824 on the right side, and he only farms 110 acres. The exodus of inhabitants from the county need not take place if the farmers could be persuaded to take a few lessons at the County Asylum. The weekly rate of maintenance is 9s. 7¼d.

### PERTH ROYAL ASYLUM.

At the beginning of the year this asylum contained 101 patients, and on the last day of December the number had fallen to 97. There were 27 admissions, 14 recoveries, and 11 deaths during the year, the percentage of recoveries standing at 41·18, and the deaths at 11·34. Hereditary influence is supposed to have caused the insanity in fourteen of the admissions, and drink is credited as the cause in one case only. Dr. Urquhart's report is, as usual, able and instructive. He touches on most things connected with asylum management, and his remarks are well put. He tells that half the cases admitted during the year were regarded as incurable, and he adds, "No doubt there are ample grounds for the oft-repeated advice urging the early treatment of insanity; but there always will be a residuum of cases that no treatment, however prompt and energetic, can benefit." This is certainly true, and when would-be promoters of hospitals for the insane talk about 60 or even 70 per cent. of cures, their silly statements merely show their ignorance of the whole question. An average of 66 per cent. of the patients were usefully employed during the year, and when we realise how difficult it often is to find employment for private patients, and how difficult it is to induce them to work when the employment has been found, we must note this eminently satisfactory result obtained by Dr. Urquhart. There would seem to have been many changes among the subordinate attendants and nurses. Such frequent changes are far from being unknown in other asylums, and although Dr. Urquhart deplores these frequent changes, he may take our word for it that they are not always an unmixed evil, and are often to be preferred to too long service. The following is worth quoting:—"It is possible that in architure and in the every-day routine of asylum management the high-water mark has been reached. It is now in the domain of scientific research and educated nursing that further progress must be made." If the high-water mark in asylum architecture has been reached in Scotland, we fear it is not more than half-tide with us in England, and while we greatly doubt whether scientific research will add much to our recovery rate in the near future, we earnestly hope that the domain of educated nursing will be an ever-widening one. The Commissioners in Lunacy say, "The patients continue to be exceedingly well provided for. They are kept in great comfort; they receive skilful treatment, and their individual requirements are carefully and kindly considered." The farm is a small one; but it is large enough to produce a favourable balance of £263. With 50 acres of land in Perth a man might jog along comfortably, and with 100 he would be rich. English farmer, there is no need to travel to the antipodes. Try Scotland.

### THE GLAMORGAN COUNTY ASYLUM, BRIDGEND.

From this, the twenty-seventh annual report, we learn that the asylum contained 940 patients on January 1st, and by the end of the year the number had risen to 970. The admissions stand at the respectable figure of 308, the recoveries at 90, and the deaths at 138. Put into percentages in the usual way, the recoveries come out at 29, and the deaths at 14·3. Various brain diseases account for 65 of the deaths, and consumption for 13, while pneumonia killed eight, and pleurisy one. Almost every professional calling seems to be represented in the admissions. Fifty-two were agricultural labourers, 30 were colliers, and 14 were shoemakers, while clergymen, music-teachers, policemen, hawkers, &c., appear by ones and twos. Table X. tells us that drink caused the insanity in 89 instances, and hereditary influence in 103. That means that two-thirds of the admissions were from causes under the direct control of man. Think of this, ye social reformers of all creeds and crazes! Here is a field big enough for you all to meet, and facts enough to give point to many a speech and many an essay! We gladly give Dr. Pringle's remarks on the new Lunacy Act a little wider publicity. After stating that it fails both as a working measure and as a protection to the insane, he proceeds as follows: "That the time of the chief medical officer of an asylum should have to be devoted to perpetual certifyings instead of to the good of the patients is as retrograde a step as could well have been made, and I am sure would never had been enacted had the constructors of the Act known anything about the duties or the practical working of an asylum. . . . I believe I am correct in saying that in the framing of this Act, experience and knowledge were regarded as disqualifications, and the counsel of those best able to give it was ignored." The farm accounts show a balance of £455 in favour of the farm, but we notice no charge for rent of land, interest on capital or patients' labour; and the prices charged to the asylum for produce supplied from the farm are not given, so that the balance-sheet might as well have been omitted. The weekly rate of maintenance is 8s. 6½d.

